# Comparative Analysis of the Chemical Constituents of *Chrysanthemum morifolium* with Different Drying Processes Integrating LC/GC–MS−Based, Non-Targeted Metabolomics

**DOI:** 10.3390/metabo14090481

**Published:** 2024-09-02

**Authors:** Na Chen, Jizhou Fan, Gang Li, Xuanxuan Guo, Xiao Meng, Yuqing Wang, Yingying Duan, Wanyue Ding, Kai Liu, Yaowu Liu, Shihai Xing

**Affiliations:** 1 Joint Research Center for Chinese Herbal Medicine of Anhui of IHM, Bozhou Vocational and Technical College, Bozhou 236800, China; 0430077002@bzy.edu.cn (N.C.); 2023130101@bzy.edu.cn (G.L.); 0430091011@bzy.edu.cn (X.G.); 0430081013@bzy.edu.cn (X.M.); 19991122wyq@stu.ahtcm.edu.cn (Y.W.); dyy@stu.ahtcm.edu.cn (Y.D.); 2 College of Pharmacy, Anhui University of Chinese Medicine, Hefei 230012, China; 17805652827@stu.ahtcm.edu.cn (J.F.); dwy111@stu.ahtcm.edu.cn (W.D.); 3 Institute of Traditional Chinese Medicine Resources Protection and Development, Anhui Academy of Chinese Medicine, Hefei 230012, China; 4 Bozhou Xinghe Agricultural Development Co., Ltd., Bozhou 236800, China; liukai@boyaohg.com; 5 Joint Research Center for Chinese Herbal Medicine of Anhui of IHM, Anhui University of Chinese Medicine, Hefei 230012, China; 6 MOE−Anhui Joint Collaborative Innovation Center for Quality Improvement of Anhui Genuine Chinese Medicinal Materials, Hefei 230038, China; 7 Anhui Province Key Laboratory of Research & Development of Chinese Medicine, Hefei 230012, China

**Keywords:** *Chrysanthemum morifolium*, non-targeted metabolome, high performance liquid chromatography, shade drying (YG), heat drying (HG)

## Abstract

*Chrysanthemum morifolium* is a perennial herbaceous plant in the Asteraceae family that is used as a medicine and food owing to its superior pharmacological properties. Irrespective of its application, *C. morifolium* must be dried before use. Shade drying (YG) and heat drying (HG) are the two drying methods used in most origins. Given the abundance of flavonoids, phenolic acids, and terpenoids, the primary medicinal active constituents of *C. morifolium*, it is important to determine whether the composition and content of these compounds are altered during the drying processes. To test this, the changes in the chemical composition of *C. morifolium* flowers after YG and HG using full-spectrum, non-targeted LC/GC–MS−based metabolomics and, subsequently, the three indicator components of *C. morifolium*—chlorogenic acid, 3,5−dicaffeoylquinic acid, and luteolin−7−O−glucoside—were accurately quantified by HPLC. The results of the non-targeted metabolomics analysis revealed that YG- and HG-processed *C. morifolium* differed significantly with respect to chemical contents, especially flavonoids, phenolic acids, and terpenoids. The levels of the indicator components and their precursors also differed significantly between the YG and HG treatments. The contents of most of the flavonoids and key phenolic acids, terpenoids, and carbohydrates were higher with YG than with HG pre-treatment. These results revealed the changes in the chemical composition of *C. morifolium* during the YG and HG processes, thus providing a reference for the further optimization of the production and processing of chrysanthemums.

## 1. Introduction

*Chrysanthemum* × *morifolium* Ramat has been used as medicine or a health-promoting ingredient since the Eastern Han dynasty. The Chinese medicine known as Juhua is the dried flower head of *C. morifolium*, and the original plants of this medicine include several strains such as Boju, Chuju, Gongju, Hangju, and Huaiju according to the processing method used [1]. *C. morifolium* has a long history of use as a traditional Chinese medicine, dating back to the Qin and Han dynasties [2]. Additionally, *C. morifolium* has a wide range of clinical applications, often used in the treatment of inflammation, headaches, hypertension, and respiratory diseases [3,4]. Recent pharmacological studies have shown that extracts of *C. morifolium* have a wide range of biological properties, such as anti-inflammatory, anti-oxidant, anti-pathogenic, anti-cancer, immunomodulatory, and hepatoprotective effects [5,6,7,8,9]. It is also frequently used in healthcare products and cosmetics, and thus has significant market value [7].

Studies have shown that flavonoids, phenolic acids, terpenoids, polysaccharides, and several essential oils are the main active ingredients in *C. morifolium* [10,11,12,13,14,15]. Among them, chlorogenic acid, 3,5−dicaffeoylquinic acid, luteolin−7−O−glucoside, 4,5−dicaffeoylquinic acid, and kaempferol−3−O−rutinoside are mainly associated with anti-oxidant activity [16,17]. Meanwhile, chlorogenic acid, 3,5−dicaffeoylquinic acid, luteolin, and luteolin−7−O−glucoside are strongly linked with the anti-inflammatory activity of *C. morifolium* [18]. Terpenoids may also play a role in the anti-inflammatory activity of *C. morifolium*, exerting an anti-asthmatic function [9]. Flavonoids in *C. morifolium* inhibit PLA2, modulate glycerophospholipid and sphingomyelin metabolic pathways, and delay the pathology of acute liver injury [8]. However, the first step in the processing of *C. morifolium*, irrespective of whether it is used as food or in cosmetics or pharmaceuticals, usually involves drying. In China, there are two drying methods for Chinese herbal *chrysanthemum* (Boju) in accordance with the existing processing standards: drying the stalks together and then cutting the stalks to preserve the flowers, or directly drying flowers, that is, shade drying (YG) and heat drying (HG). They are widely used to process *C. morifolium*, however, fresh flowers and stalks are not utilized for medicinal purposes. There is, however, no standard to judge the merits of heat drying and shade drying, so we want to explore the effect of two processing methods on the quality of *C. morifolium* (boju). In this study, we compared the differences and changes in the chemical composition of *C. morifolium* under two different drying processing methods using non-targeted metabolomic analysis and high-performance liquid chromatography (HPLC). Furthermore, we quantified three compounds of *C. morifolium* that may have pharmacological activity based on previous studies conducted [18], aiming to identify a suitable pre−treatment method for the drying of *C. morifolium*, as well as providing a reference for its further processing and utilization.

## 2. Materials and Methods

### 2.1. Chemicals and Plant Materials

The *C. morifolium* plants used in this study were cultivated by Bozhou Vocational and Technical College and identified by Associate Prof. Qingshan Yang of Anhui University of Traditional Chinese Medicine as Boju in the Compositae family. The same shape and size of *C. morifolium* (Boju) flowers were selected from the same field when the 12 groups were sampled separately. For 6 of these groups (HG 1–6), the flowers were collected directly from the heads and dried in an oven at 55 °C. For the remaining 6 groups (YG 1–6), the flowers were collected together with the stalks (approximately 15 cm), hung in a cool and ventilated place for 30 days, and then the flowers were collected. All the samples were rapidly vacuum freeze−dried and then pulverized to a powder, passed through a 50-mesh sieve, and immediately stored at −80 °C. All the chemicals and solvents used were of analytical or HPLC grade (Supplementary Table S1).

### 2.2. Sample Preparation

For the non-targeted metabolomic analysis, a 40 mg sample was precisely weighed and transferred into a 1.5-mL centrifuge tube, to which two small steel beads and 800 μL of methanol and water (7:3 [*v*/*v*], with 2−chloro−L−phenylalanine, succinic acid−2,2,3,3−d4, L−valine−d8, and cholic acid−d4 as a mixed internal standard, 4 μg/mL) were added. The samples were pre−cooled at −40 °C for 2 min, ground in a grinder (Organic phase nylon needle filter, green, Shanghai, China) for 2 min (60 Hz), extracted by ultrasonication in an ice−water bath for 30 min, and left to stand at −40 °C overnight. The next day, the samples were centrifuged at 12,000 rpm for 10 min at 4 °C, and 150 μL of the supernatant was passed through a 0.22-μm organic filter membrane, transferred to an LC injection vial, and finally stored at −80 °C until used for LC–MS analysis. Another 150 μL of the supernatant was centrifuged and evaporated to dryness, followed by the addition of 80 μL of pyridine methoxylamine hydrochloride solution (15 mg/mL), and the oximization reaction was carried out for 60 min at 37 °C in a shaking incubator. Subsequently, 50 μL of BSTFA derivatization reagent, 20 μL of hexane, and 10 μL of 10 internal standards (methyl octanoate, methyl nonanoate, methyl decanoate, methyl dodecanoate, methyl tetradecanoate, methyl hexadecanoate, methyl octadecanoate, methyl eicosanoate, methyl docosanoate, and methyl xxivoate, all of which were configured with chloroform) were added and allowed to react at 70 °C for 60 min. After leaving it to stand at room temperature for 30 min, GC–MS metabolomics analysis was performed. Quality control (QC) samples were prepared by mixing equal volumes of extracts from all the samples, and this mixture was used to equilibrate the mass spectrometer.

### 2.3. Metabolomics Analysis Based on LC–MS

A Waters ACQUITY UPLC I−Class plus/Thermo QE HF ultra−high performance liquid chromatography–tandem high-resolution mass spectrometer was used for the analysis. Chromatographic separation was performed on an ACQUITY UPLC HSS T3 (100 × 2.1 mm, 1.8 µm) (Waters, Milford, MA, USA) column at 45 °C. The mobile phase consisted of (A) 0.1% formic acid in water (*v*/*v*) and (B) acetonitrile, the flow rate was 0.35 mL/min, and the injection volume was 3 μL. The optimal gradient elution program was as follows: 0~2 min, 5% B; 2~4 min, 30% B; 4~8 min, 50% B; 8~10 min, 80% B; 10~14 min, 100% B; 14~15 min, 100% B; 15~15.1 min, 5% B; and 15.1~16 min, 5% B. To analyze the metabolic profiles, mass spectrometry was performed in positive electrospray ionization (ESI) mode and negative ESI mode under the conditions shown in Table 1. The QC samples were injected every six samples throughout the analysis for the assessment of reproducibility and for evaluating the stability of the mass spectrometer system during the sample detection.

### 2.4. Metabolomics Analysis Based on GC–MS

An Agilent 8890−5977B Gas chromatograph-mass spectrometer was used for the GC–MS analysis. Gas chromatography was performed on a DB−5MS capillary column (30 m × 0.25 mm × 0.25 μm, Agilent J&W Scientific, Folsom, CA, USA). High−purity (≥99.999%) helium served as the carrier gas. The flow rate was 1 mL/min, the temperature at the injection port was 260 °C, the injection volume was 1 μL, and the sample was injected without shunt. The initial temperature of the column oven was 60 °C and was held for 0.5 min, the temperature was increased to 125 °C at 8 °C/min, and then to 210 °C at 8 °C/min, 270 °C at 15 °C/min, and 305 °C at 20 °C/min, and held for 5 min. The gas−phase mass spectrometric analysis was performed using an electron bombardment ion source with an ion−source temperature of 230 °C, a quadrupole temperature of 150 °C, and an electron energy of 70 eV. Scanning was performed in full−scan mode, and the mass scanning range was *m*/*z* 50–500. QC samples were injected every six samples throughout the analysis.

### 2.5. LC/GC–MS Data Analysis

Before further analysis, the LC–MS and GC–MS results were first processed using Progenesis QI v3.0 and MS−DIAL software (v4.24) to obtain the initial data matrix. Metabolites were identified based on multiple dimensions such as retention time, exact mass, secondary fragmentation, and isotopic distribution. The Human Metabolome Database (HMDB), Lipidmaps (v2.3), the METLIN database, the LuMet−Plant 3.0 local database (Oebiotech Company, Shanghai, China), and the LuMet−GC 5.0 database (untargeted GC–MS database from Lumingbio) were used for metabolite identification and analysis [19]. To visualize the metabolic differences between the YG and HG methods, Principal Component Analysis (PCA) and Orthogonal Partial Least Squares−Discriminant Analysis (OPLS−DA) were performed based on the LC–MS and GC–MS data using an online analytical method (https://www.metaboanalyst.ca/, accessed on 3 November 2023). Meanwhile, to prevent overfitting, the quality of the model was examined by seven−fold cross−validation and 200 response permutation testing (RPT). The detailed analysis process was per our previous reports [20,21].

The significance of the differences between samples was defined as Variable Importance in Projection (VIP) values > 1 in the OPLS−DA model analysis and statistically significant *p*-values (<0.05) from a Student’s *t*-test (two-tailed) of peak areas in the different groups. Differential metabolites were visualized using volcano plots and heatmaps. At the same time, the differential metabolites were mapped to KEGG pathways, and the number of metabolites enriched in the corresponding pathways was counted [22]. Pathways with a *p*-value ≤ 0.05 were considered significantly enriched [19].

### 2.6. High−Performance Liquid Chromatography

HPLC analysis was performed using the Agilent 1260 Infinity II LC System (Agilent Technologies, Santa Clara, CA, USA). The chromatographic column was a ZORBOX SB−C18 column (250 × 4.6 mm, 5 μm, Agilent) and the column temperature was 35 °C. The gradient elution system consisted of acetonitrile (A) and 0.1% phosphoric acid in water (B). The gradient elution program was as follows: 0~11 min, 10~18% A; 11~30 min, 18~20% A, held for 20 min. The method was effective at determining linearity, precision, stability, and repeatability. The results are summarized in Supplementary Table S2. Data were analyzed using IBM SPSS v.23. One-way analysis of variance (ANOVA) was applied to determine differences among samples; statistical differences were compared using *t*−tests. Finally, graphing was performed in GraphPad Prism 8.0.1.

## 3. Results

### 3.1. Identification of the Global Metabolite Profile Based on GC–MS and LC–MS Metabolomics

**Figure 1:**
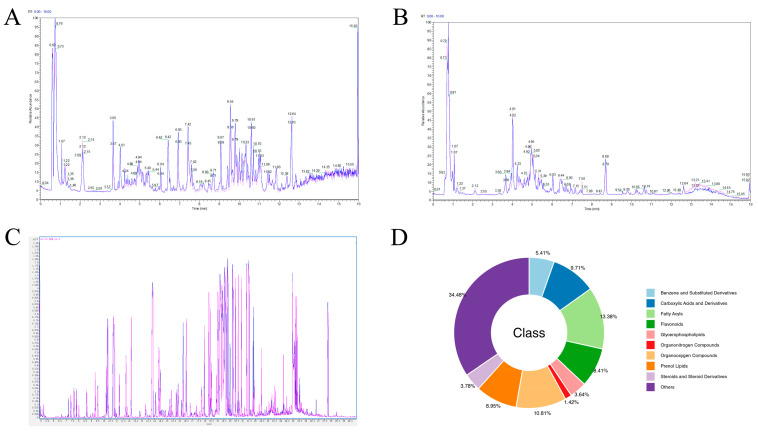
The global metabolite profile of Chrysanthemum morifolium flowers based on gas chromatography–mass spectrometry (GC–MS) and liquid chromatography–mass spectrometry (LC–MS). (**A**) LC–MS chromatograms in positive mode electrospray ionization (ESI). (**B**) LC–MS chromatograms in negative mode ESI. (**C**) GC–MS chromatograms. Blue, shade-drying group (YG); red, heat−drying group (HG). (**D**) The superclass of 7908 metabolites identified. Different color blocks represent different compound classes.

LC–MS and GC–MS were used to perform non-targeted metabolomic measurements on HG and YG samples to identify the major differential metabolites of *C. morifolium* between these two processes. The LC−MS chromatogram is shown in Figure 1A,B and the GC–MS chromatogram is depicted in Figure 1C. A total of 20,831 substance peaks were detected in the YG and HG samples by LC–MS, with 7735 metabolites being identified, including 3495 in negative ion mode (ESI−) and 4110 in positive ion mode (ESI+). A total of 173 substance peaks were detected by GC–MS in both the YG and HG samples, all of which were annotated as metabolites. All metabolite information is provided in Supplementary Table S3. The 7908 metabolites identified were categorized according to the classification criteria in the HMDB database and the nine classes with the highest numbers of metabolites are shown in Figure 1D. Most of the identified species were fatty acyls (13.38%), organooxygen compounds (10.81%), carboxylic acids and derivatives (9.71%), prenol lipids (8.95%), flavonoids (8.41%), benzene and substituted derivatives (5.41%), and glycerophospholipids (3.64%).

### 3.2. Multivariate Analysis of the Identified Metabolites

**Figure 2:**
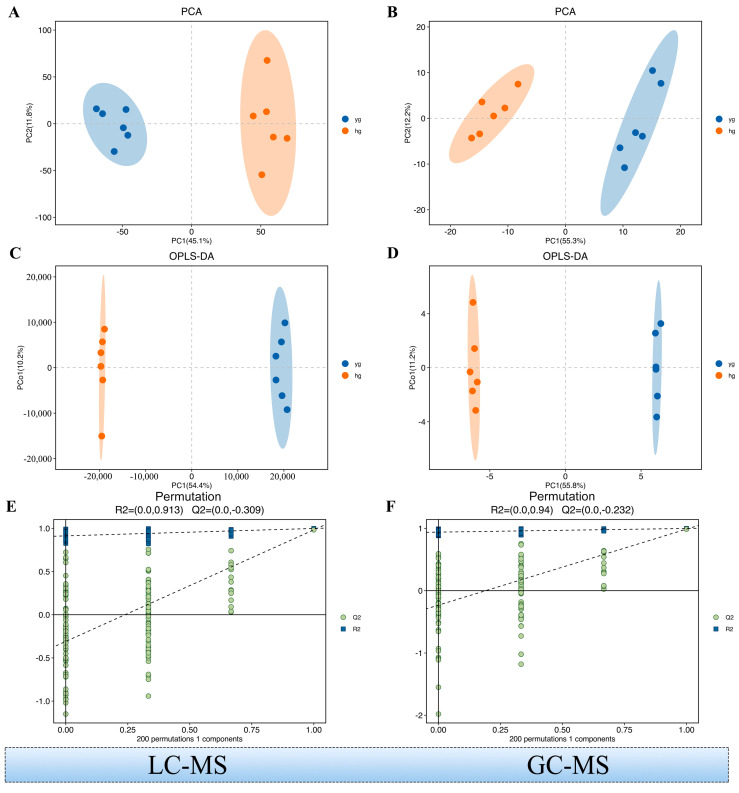
Multivariate model and its cross-validation. (**A**,**B**) Principal Component Analysis (PCA) of the LC/GC–MS data. (Combine both positive and negative ion data to generate) (**C**,**D**) Orthogonal Partial Least Squares−Discriminant Analysis (OPLS−DA) of the LC/GC–MS data. (**E**,**F**) Response permutation testing of the model predicted by OPLS−DA. R2X (cum): cumulative interpretation rate in the X direction; R2Y (cum): cumulative interpretation rate in the Y direction; Q2 (cum): cumulative forecast rate of the model; R2 and Q2: parameters of the response sequencing test used to measure whether the model was overfitted.

We first performed a PCA, an unsupervised data analysis method, on the 7908 identified metabolites to observe the overall distribution trend among the samples and identify the inter- and intra-group variability. There was a marked separation of metabolites between the YG and HG groups (Figure 2), indicating that they were significantly different. Meanwhile, OPLS−DA, a supervised method, was used to maximally highlight the differences in the metabolite profiles between the two groups of samples, with the R2X and Q2 values indicating good model quality (Figure 2C).

### 3.3. Screening and Classification of the Differential Metabolites 

**Figure 3:**
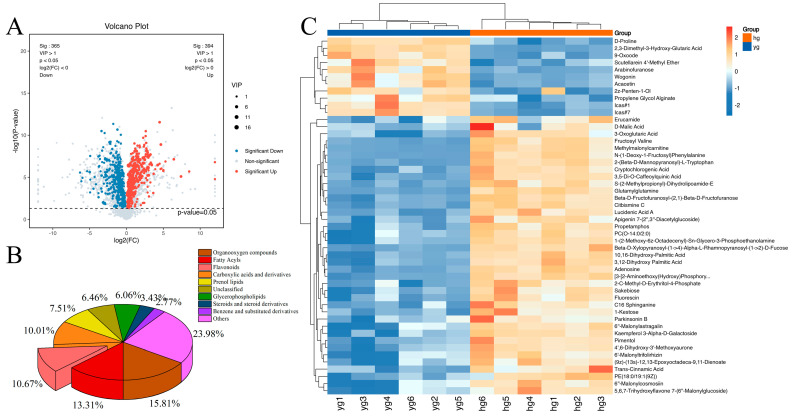
Differentially abundant metabolites between the YG and HG samples. (**A**) A volcano plot comprising both LC–MS and GC–MS data of the 7908 metabolites identified by metabolomics analysis. VIP > 1 and *p* < 0.05 served as the criteria for differential metabolite classification. (**B**) Classification of the 759 differential metabolites. (**C**) A heat map of top 50 differential metabolites. (VIP: Variable importance in the projection. P: Probability Value. HG: heat drying. YG: Shade drying).

We selected the metabolites with VIP values > 1 and *p*-values < 0.05 as differential metabolites (Figure 3). A volcano plot of the upregulated and downregulated metabolites is shown in Figure 3A (HG versus YG). After screening, a total of 759 differential metabolites were identified between the YG and HG samples. In total, 394 metabolites were upregulated while 365 were downregulated in the HG samples relative to the YG samples. Additionally, we categorized these 759 differential metabolites based on the HMDB database (the nine categories with the highest number of metabolites were selected) and found that they comprised mainly organooxygen compounds (15.81%), fatty acyls (13.31%), flavonoids (10.67%), carboxylic acids and derivatives (10.01%), and prenol lipids (7.51%) (Figure 3B). Based on the VIP values, we further screened the top fifty of the 759 differential metabolites and plotted a heat map (Figure 3C and Supplementary Table S4). Heat map analysis results indicated that there was a significant difference in the abundance of these compounds between the YG and HG groups.

### 3.4. Determination of YG and HG Indicator Active Ingredients by HPLC

**Figure 4:**
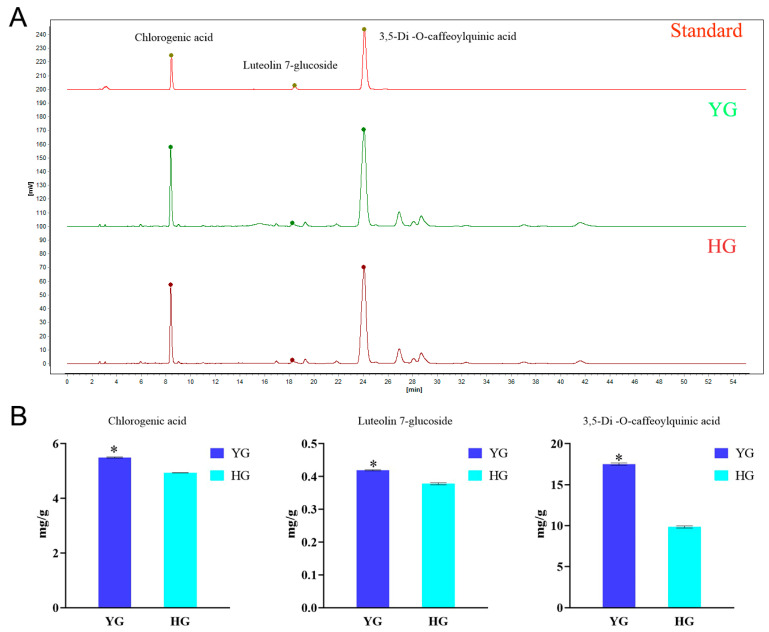
High-performance liquid chromatography (HPLC) analysis of 3 medicinal ingredients in *chrysanthemum* (boju). (**A**) HPLC chromatogram for 3 medicinal ingredients in *chrysanthemum* (boju). (**B**) Bar charts of the three indicator components of *chrysanthemum* (boju) (* *p* < 0.05).

To accurately quantify the main active components of *C. morifolium* prepared using the YG and HG methods, we quantified the contents of chlorogenic acid, 3,5−O−dicaffeoyl−quinic acid, and luteolin−7−O−glucoside in YG and HG samples by HPLC (Figure 4A and Figure S1). We found that the concentrations of these three metabolites in the YG samples were 5.4984, 17.5193, and 0.4189 mg/g, respectively, and in the HG samples were 4.9367, 9.8494, 0.3779 mg/g, respectively, (Figure 4B). The contents of chlorogenic acid, luteolin−7−O−glucoside, and quinic acid were significantly higher in the YG samples than in the HG samples. The greatest difference was found in quinic acid levels, with the YG samples having approximately 1.78-fold the quinic acid content of the HG samples. However, compared with the Chinese Pharmacopoeia, the content of luteolin−7−O−glucoside in both samples is relatively small.

### 3.5. KEGG Classification and Enrichment Analysis of the Differential Metabolites

**Figure 5:**
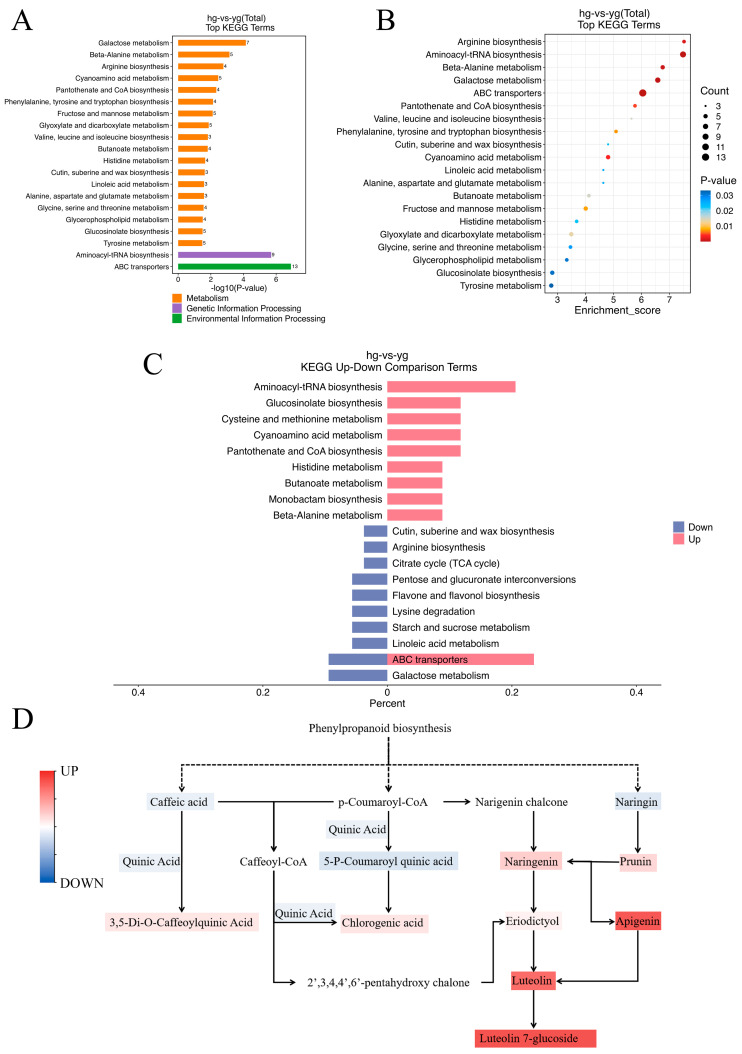
Kyoto Encyclopedia of Genes and Genomes (KEGG) pathway enrichment analysis. (**A**) The top 20 pathways in terms of −lg (*p*-value) for the HG samples compared to the YG samples. (**B**) A KEGG analysis bubble plot of the top 20 enriched pathways. (**C**) The 10 most significantly (smallest *p*-value) upregulated and downregulated KEGG metabolic pathways between the HG and YG groups. (**D**) The chlorogenic acid, 3,5−O−dicaffeoyl−quinic acid, and luteolin−7−O−glucoside synthetic pathways.

To reveal the metabolic pathway differences among the metabolites displaying differential abundance between the YG and HG samples, the differential metabolites were submitted to KEGG metabolic pathway enrichment analysis. Figure 5A shows the top 20 enriched pathways in terms of −lg (*p*-value) for the HG samples versus the YG samples. A KEGG analysis bubble plot reflecting the amounts of metabolites, enrichment analysis scores, and *p*-values for each pathway is shown in Figure 5B. Meanwhile, we plotted the comparison of the 10 most significantly (smallest *p*-value) upregulated and downregulated KEGG metabolic pathways between the HG and YG groups (Figure 5C). Compared with the YG samples, the 9 most significantly upregulated pathways (smallest *p*-value) in the HG samples were Amino sugar and nucleotide sugar metabolism, Glucosinolate biosynthesis, Cysteine and methionine metabolism, Cyanoamino acid metabolism, Pantothenate and CoA biosynthesis, Histidine metabolism, Butanoate metabolism, Monobactam biosynthesis, and beta-Alanine metabolism. Meanwhile, the 9 most significantly upregulated pathways in the HG samples relative to the YG samples were Cutin, suberine and wax biosynthesis, Arginine biosynthesis, Citrate cycle (TCA cycle), Pentose and glucuronate interconversions, Flavone and flavonol biosynthesis, Lysine degradation, Starch and sucrose metabolism, Linoleic acid metabolism, and Galactose metabolism.

Among the 20 pathways included in Figure 5A, Phenylalanine, tyrosine and tryptophan biosynthesis underlies the synthesis of chlorogenic acid, 3,5−O−dicaffeoyl−quinic acid, and luteolin−7−O−glucoside, which suggests that the YG and HG methods have a strong influence on the retention of the indicator active constituents of *C. morifolium*. Thus, we mapped the biosynthetic pathways for these three metabolites via the literature and database searches (Figure 5D) and then assessed the changes in the levels of components related to chlorogenic acid, 3,5−O−dicaffeoyl−quinic acid, and luteolin−7−O−glucoside in the YG and HG samples by measuring the metabolome peak area, as well as using HPLC quantification. We found that both the YG and HG *C. morifolium* samples experienced a significant decrease in all three indicator components. Among them, the contents of caffeic acid, quinic acid, and 5−P−coumaroylquinic acid, the precursors of 3,5−O−dicaffeoyl−quinic acid, and chlorogenic acid were higher in the HG samples than in the YG samples, whereas 3,5−O−dicaffeoyl−quinic acid and chlorogenic acid showed the opposite trend. This suggested that *C. morifolium* processed by the HG method may have lost a significant amount of 3,5−O−dicaffeoyl−quinic acid and chlorogenic acid in the final production. The levels of most of the precursors in the luteolin−7−O−glucoside synthetic pathway were lower in the HG samples than in the YG samples, as were those of luteolin−7−O−glucoside.

## 4. Discussion

The active components and medicinal value of *Chrysanthemum*, in particular its anti-inflammatory and immunomodulatory effects, and improved hyperlipidemia [23], have been extensively studied [24,25]. In addition, *Chrysanthemum* has shown potential ameliorative effects in complex conditions such as chronic metabolic diseases and neurodegenerative disorders, which further highlights its value for applications in the fields of medicine and healthcare. An in−depth analysis showed that flavonoids, phenolic acids, and terpenoids were the major active components of *Chrysanthemum*, which may underlie its above-mentioned effects [26,27,28]. The YG and HG processing methods may exert significant effects on the chemical composition and bioactivities of *Chrysanthemum*. In the drying process, some heat-sensitive compounds may be lost or transformed. To obtain a more comprehensive understanding of the effects of these treatments on *Chrysanthemum* quality, we conducted an in-depth analysis of the flavonoids, phenolic acids, and other active compounds identified among the differential metabolites.

### 4.1. The YG Samples Had a Higher Flavonoid Content than the HG Samples, Which May Endow C. morifolium with Better Anti-Oxidant Activity

**Figure 6:**
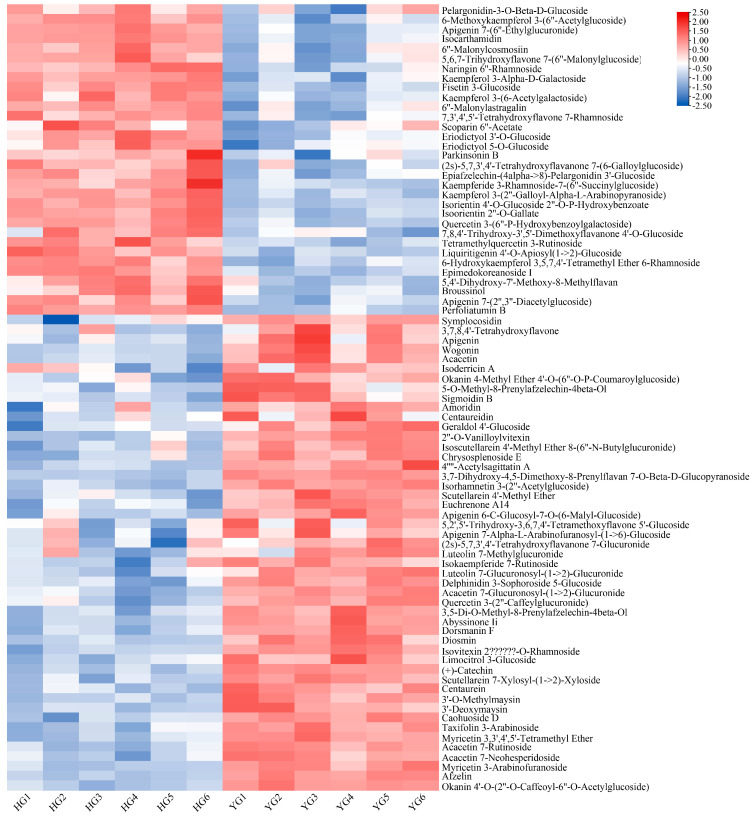
A heatmap of the 81 flavonoids in HG− and YG−processed *C. morifolium*.

Flavonoids, especially their glycosides, are considered the major active compounds of *C. morifolium*, and Total flavonoids of Chrysanthemum have been proved to inhibit inflammation and apoptosis [29]. Flavonoids, such as lignans, apigenin, diosgenin, and vinpocetine were found to be the most abundant metabolites in *C. morifolium*, followed by flavonols and flavanones, including quercetin, rutin, hesperidin, and their derivatives [30]. In the KEGG analysis, Flavone and flavonol biosynthesis showed significant downregulation in the HG samples. One study showed that differences in total flavonoid contents in different *C. morifolium* varieties lead to significantly different anti-oxidant activities [31]. Moreover, a different study reported that the contribution of flavonoids to the anti-oxidant activity of *C. morifolium* was in the order quercetin > luteolin > acacetin > apigenin > hesperetin [32]. In plants, flavonoids play a role in protection against ultraviolet radiation and defense against pathogens and herbivores. Additionally, they are widely used as anti-oxidants and colorants in pharmaceuticals, food, and cosmetics [33]. Many studies have shown that the intake of isorhoifolin, diosmin, and linarin helps to reduce the risk of several chronic diseases and cancers [34], and all three of these flavone rutinosides are enriched in *C. morifolium* [35,36]. We detected a total of 665 flavonoids in *C. morifolium*, 81 of which were significantly altered in YG- and HG-processed *C. morifolium* (Figure 6 and Supplementary Table S5). These 81 flavonoids included acetin, apigenin, their derivatives, luteolin derivatives, quercetin derivatives, and diosmin, among others. Although the contents of quercetin derivatives were higher in HG samples than in YG samples, the abundance of most flavonoids, such as apigenin, acetin, aiosmin, their derivatives, and luteolin derivatives, was greater in YG-processed *C. morifolium* than in HG-treated samples. The higher levels of flavonoids in the YG group compared with the HG group may endow *C. morifolium* with better anti-oxidant activity, although studies on the strong anti-oxidant effect of chrysanthemum flavonoids have been reported [37]. The possibility that the YG may have better anti-oxidant effects needs to be verified by further pharmacological experiments.

### 4.2. YG Treatment Resulted in Higher Levels of Phenolic Acids, Which May Endow C. morifolium With Better Anti-Oxidant Activity

Phenolic acids, organic acids containing phenolic rings, are the active ingredients responsible for the antipyretic, analgesic, and anti-oxidant effects of *C. morifolium*. The phenolic acids found in *C. morifolium* include caffeic acid, ferulic acid, chlorogenic acid, 3,5−O−dicaffeoyl−quinic acid, 3,4,5−O−tri−caffeoyl−quinic acid, ethyl caffeate, and its isomers. Chlorogenic acid and 3,5−O−dicaffeoyl−quinic acid have a significant impact on the anti-oxidant capacity of *C. morifolium*.

Most of the caffeoylquinic acid in the hot water extract of *C. morifolium* is dissolved within 30 min [30], and its hydrolysis products may react with glycosides or glycosyl groups to form a green substance, resulting in a tea broth with yellowish-green color [38]. Chlorogenic acid can be used in the treatment of diabetes, cardiovascular disease, neurodegenerative diseases, and cancer. It is also an excellent candidate as a natural dietary additive and for use in functional food formulations owing to its favorable antimicrobial and anti-oxidant activities [39]. In the Chinese Pharmacopoeia, the contents of chlorogenic acid, 3,5−O−dicaffeoyl−quinic acid, and luteolin−7−O−glucoside are used as quality measures for *C. morifolium*−containing herbs [40]. Although the three compounds alone cannot completely represent the quality of the Chinese herbal medicine, juhua, the contents of these three compounds are nonetheless of great reference value for quality evaluation [30]. Here, we found that the contents of chlorogenic acid, 3,5−O−dicaffeoyl−quinic acid, and luteolin−7−O−glucoside were higher in the YG samples than in the HG−processed ones, which indicates that the quality of *C. morifolium* after YG treatment might be better than after HG treatment.

### 4.3. Other Active Compounds That Were Higher in the YG Group than in the HG Group

**Figure 7:**
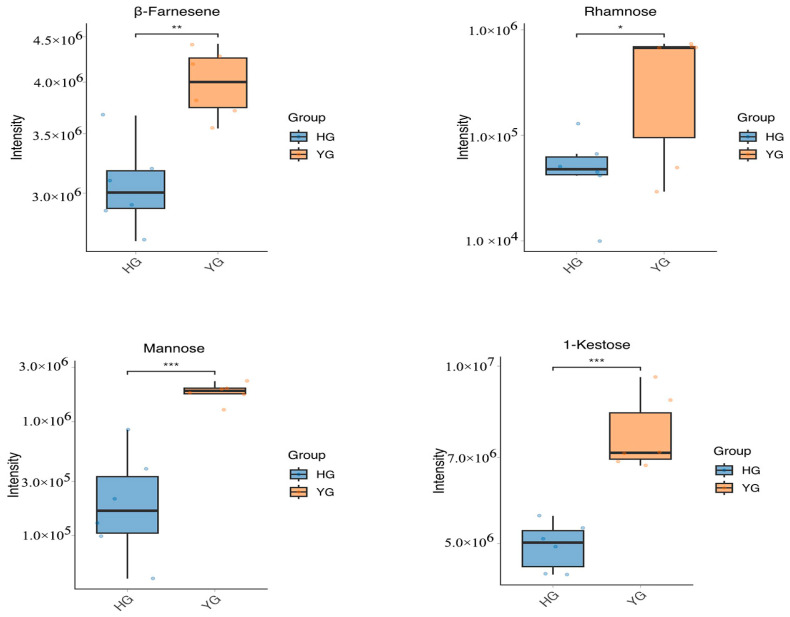
Box plots of β-farnesene, mannose, rhamnose, and 1-kestose. (* *p* < 0.05; ** *p* < 0.01; *** *p* < 0.001).

Volatile oils constitute the main material basis for the release of aromatic odors from *C. morifolium*, which is one reason why *C. morifolium* is used in perfumes and as a floral tea. Kuang et al. [41] identified 56 compounds in the extracted essential oil of *C. morifolium*, most of which were monoterpenes and sesquiterpenes. Jiang et al. [42] identified monoterpenes in *C. morifolium*, mainly including camphor, eucalyptol, bornyl acetate, and β-myrcene, as well as sesquiterpenes, including γ-selinene, β-farnesene, β-elemene, β-car-yophyllene, and β-curcumene. *C. morifolium* polysaccharides, primarily consisting of glucose, galactose, mannose, arabinose, rhamnose, and galacturonic acid are also important medicinal ingredients and are associated with the flavor of *C. morifolium* [43]. Notably, *Chrysanthemum* leaves, buds, and blooms contain high levels of raffinose and 1-kestose [44]. These natural sugars not only give *Chrysanthemums* their distinctive sweet flavor but also provide numerous health benefits. Other monoterpenes, oligosaccharides, and polysaccharides were not significantly different. We found that β-farnesene, mannose, rhamnose, and 1-kestose contents were also significantly different between the samples treated using the two processing methods, with the YG samples exhibiting higher concentrations of these metabolites compared with the HG samples (Figure 7 and Supplementary Table S6). This suggests that the color, odor, and taste of *C. morifolium* may also be superior with YG treatment than with HG treatment.

## 5. Conclusions

We found that the HG and YG methods of processing *C. morifolium* resulted in a significant difference in quality. Using metabolomic analysis, we noted that flavonoids, phenolic acids, and some other active compounds were significantly more abundant with YG treatment than with HG treatment. In accordance with the Chinese Pharmacopoeia, we accurately quantified the contents of chlorogenic acid, 3,5−O−dicaffeoyl−quinic acid, and luteolin−7−O−glucoside, which are the indicator components of *C. morifolium*, and found that the abundance of these three constituents was also significantly higher in YG-treated samples than in HG-processed ones. These results suggest that the quality of YG-processed *C. morifolium* may be superior to that of HG-processed *C. morifolium*; our findings further indicate that the quality of *C. morifolium* may be improved by adopting the YG approach for pre−treatment. However, it has not been experimentally verified whether the differences in components lead to variations in the efficacy of YG and HG. We plan to do further pharmacological experiments of anti-oxidant activity of *Chrysanthemum morifolium (Boju).*

## Figures and Tables

**Table 1:** Mass spectrometry parameters of LC–MS.

Parameters	Positive Ion Mode	Negative Ion Mode
Spray voltage (V)	3800	−3000
Capillary temperature (°C)	320	
Auxiliary gas heater temperature (°C)	350	
Sheath gas flow rate (arb)	35	
Auxiliary gas flow rate (arb)	8	
S−lens RF level	50	
Mass range (*m*/*z*)	70–1050	
Full MS resolution	60,000	
MS/MS resolution	15,000	
NCE/stepped NCE	10, 20, 40	

RF, radio frequency; NCE, normalized collision energy.

## Data Availability

The original contributions presented in the study are included in the article/Supplementary Materials; further inquiries can be directed to the corresponding authors.

## Supplementary Materials

The following supporting information can be downloaded at: https://www.mdpi.com/article/10.3390/metabo14090481/s1, Supplementary Figure S1: Standard curves of three indicator components; Supplementary Figure S2: Heat drying C. morifolium; Supplementary Figure S3: Shade drying C. morifolium; Supplementary Table S1: The informationof all chemicals and solvents Supplementary Table S2: Regression curves, precision, repeatability and stability of three indicator components; Supplementary Table S3: The information of 7908 metabolites; Supplementary Table S4: The information of top fifty differential metabolites; Supplementary Table S5: The information of 81 flavonoids; Supplementary Table S6: The information of β-farnesene, mannose, rhamnose and 1-Kestose.
