# Supplementary figures and images for: Comparative Analysis of the Chemical Constituents of Chrysanthemum morifolium with Different Drying Processes Integrating LC/GC–MS−Based, Non-Targeted Metabolomics
Source: Metabolites. 2024 Sep 2;14(9):481. doi: 10.3390/metabo14090481 (PMC11434334; doi:10.3390/metabo14090481)

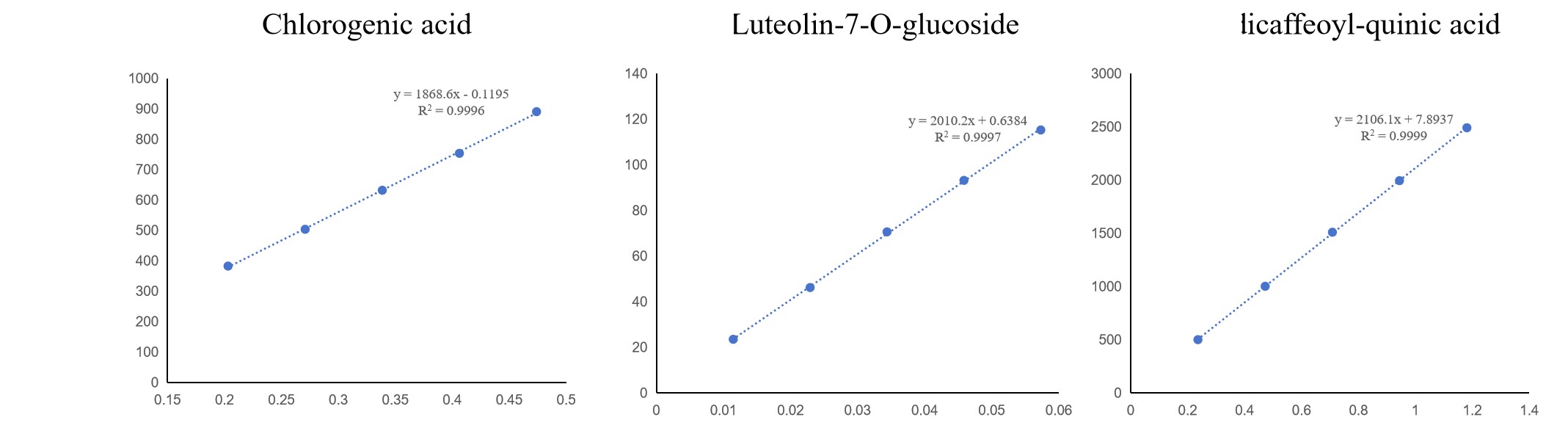

Supplement: Supplementary file 1 [file metabolites-14-00481-s001.zip › Supplementary Figure S1.Standard curves of three indicator components.jpg]

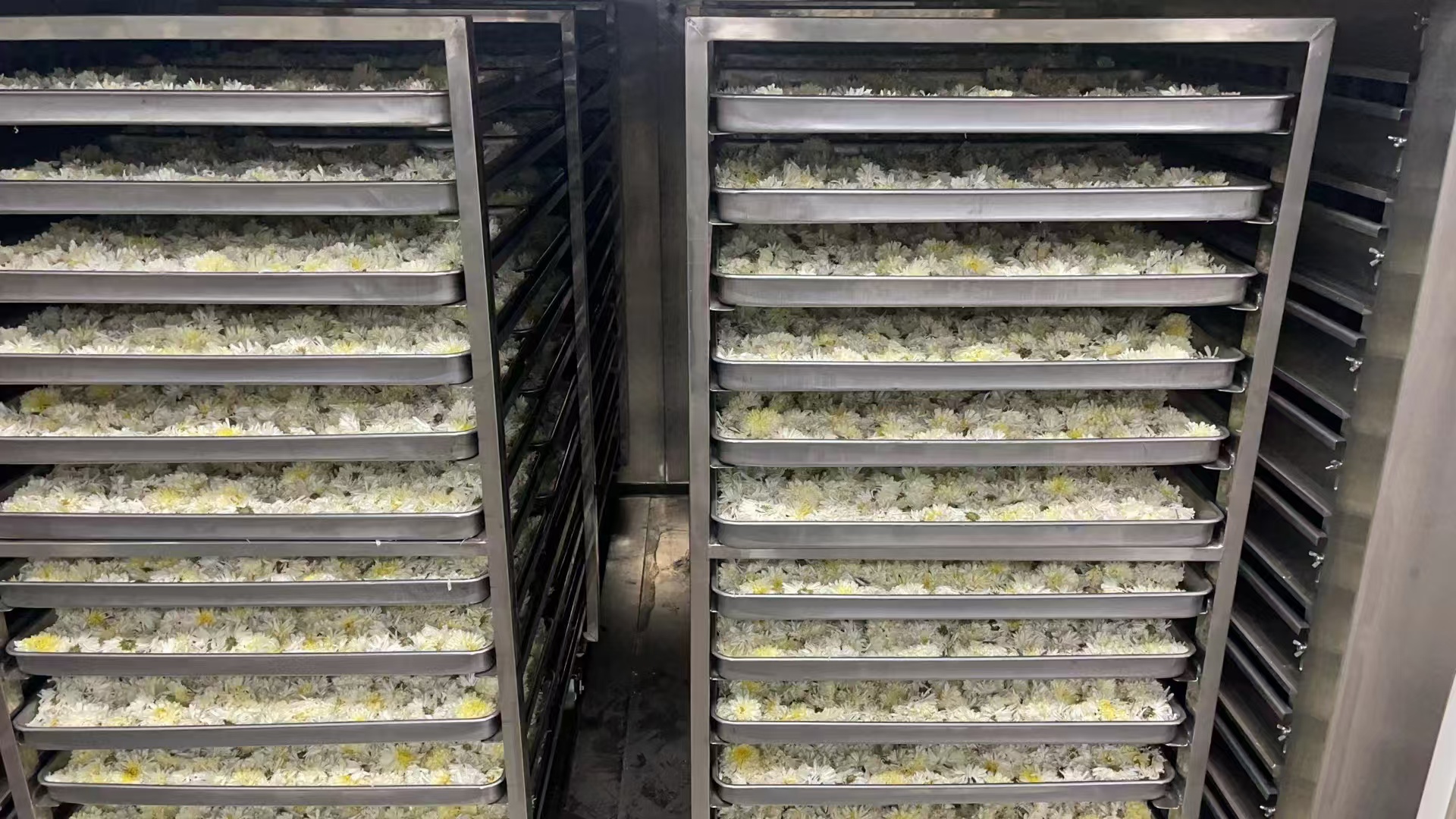

Supplement: Supplementary file 1 [file metabolites-14-00481-s001.zip › Supplementary Figure S2.Heat drying C. morifolium.jpg]

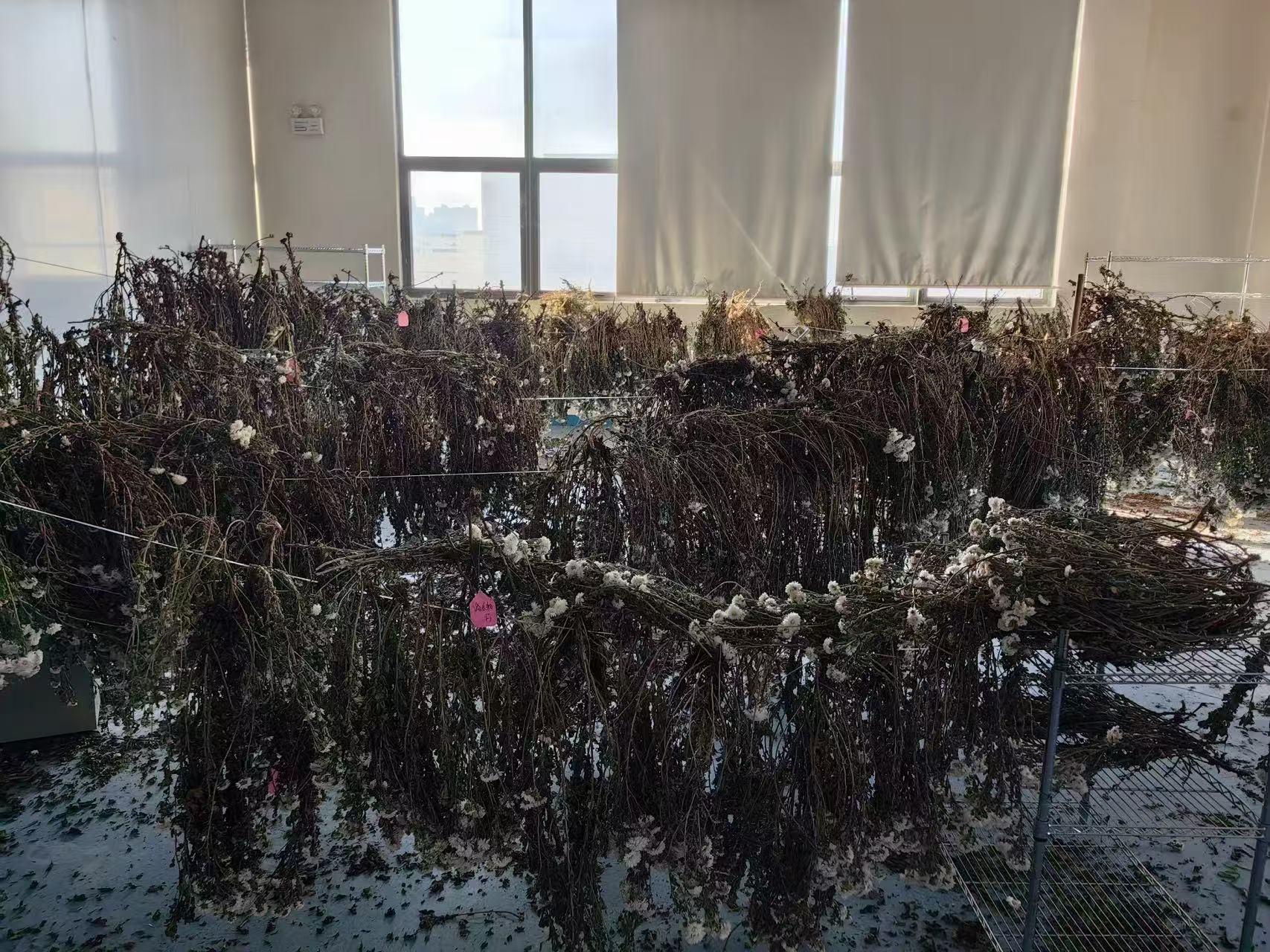

Supplement: Supplementary file 1 [file metabolites-14-00481-s001.zip › Supplementary Figure S3. Shade drying C. morifolium.jpg]
